# Incidence of Colorectal Cancer in Selected Countries of Latin America: Age-Period-Cohort Effect

**DOI:** 10.31557/APJCP.2020.21.11.3421

**Published:** 2020-11

**Authors:** Thayana Calixto de Carvalho, Anne Karin da Mota Borges, Ilce Ferreira da Silva

**Affiliations:** 1 *National Public Health School, Oswaldo Cruz Foundation, Rio de Janeiro, Brazil. *; 2 *National Cancer Institute José Alencar Gomes da Silva, Rio de Janeiro, Brazil. *; 3 *Department of Epidemiology and Quantitative Methods, National Public Health School, Oswaldo Cruz Foundation, Rio de Janeiro, Brazil. *

**Keywords:** Colorectal neoplasms, age effect, period effect, cohort effect

## Abstract

**Objective:**

To estimate Age-Period-Cohort effects on colorectal, colon and rectal cancer incidence rates in Latin American countries covered by high quality Population-Based Cancer Registries.

**Methods:**

A trend study was performed using data from Cancer Incidence in Five Continents. Age-Period-Cohort effects were estimated by Poisson regression for individuals aged between 20 and 79 years with colorectal, colon and rectal cancers informed by Population-Based Cancer Registries from 1983 to 2012 in Cali (Colombia); from 1983 to 2007 in Costa Rica; and from 1988 to 2012 for both Goiânia (Brazil) and Quito (Ecuador). Goodness of fit model was tested using the deviance of the models.

**Results:**

Age effect was statistically significant for both sexes in all Population-Based Cancer Registries areas and the curves slope reached peaks in the older age groups. There were cohort effects on the incidence rates for colorectal, colon and rectal cancers in all Population-Based Cancer Registries areas, except for women in Quito. Regarding the period effect, an increased ratio rate was observed in men (1.26, 95%CI 1.17 to 1.35) and women (1.23, 95%CI, 1.15 to 1.32) in Goiânia, between 2003 and 2007.

**Conclusions:**

In Latin America, age effect was observed on incidence rates for colorectal, colon and rectal cancers. Besides, birth cohort effect was identified for recent cohorts in both genders for colorectal, colon and rectal cancers in Cali and Goiânia, and cohort effect for colorectal and colon cancers in both genders in Costa Rica; while in Quito a cohort effect was only observed for rectal cancer among men. Period effect was observed in Goiânia with increased ratio rate in 2003-2007.

## Introduction

Colorectal cancer distribution varies considerably worldwide according to regions and age groups. Higher incidence rates are observed in developed countries, while developing countries such as Latin America’s, incidence rates have been increasing remarkably in the past 25 years (Sierra and Forman, 2016; Bray et al., 2018). In one hand, this increase could be explained by demographic transition, extended access to health care, screening programs, improvement of social-economic indexes, and lifestyle changes that eventually led to a major exposure to risk factors (Tsoi et al., 2017). On the other hand, the specific reasons affecting trends trends in Latin America countries are still unclear.

Trend analysis of cancer incidence and/or mortality are often performed through age-adjusted rates while the other effects are ignored (Latorre and Cardoso, 2001). Evidences have shown that the risk for colorectal cancer increases progressively from 40 years and steeply after 50 years old (Arnold et al., 2017; Siegel et al., 2017). Beyond age effect, some events occurred in certain periods might affect throughout age ranges (period effect). Likewise, factors can affect a generation as well as promote distinct changes in successive age groups and periods (cohort effect) (Holford, 1991). Therefore, the APC (age-period-cohort) models allow to estimate the age, period and birth cohort effect, determining which one of them impacted more in the incidence and mortality rates (Holford, 1991).

Studies examining the APC effects showed a cohort effect in the increase of colorectal cancer incidence rates in the last five decades worldwide (de Kok et al., 2008; Larsen and Bray, 2010), suggesting an increasing exposure to risk factors in successive birth cohorts(de Kok et al., 2008). Nonetheless, APC studies are mainly carried out in developed countries reflecting a different reality when compared to developing countries, since the associated factors prevalence as well as access to health care services differ between countries according to development levels (Arnold et al., 2017). In developing countries, the paucity of studies is partly explained by the fact that an organized Population-Based Cancer Registry (PBCR) with time series of uninterrupted high quality information is a requirement. Among the active and existing PBCR in Latin America, only registries located in Goiânia (Brazil), Quito (Ecuador), Cali (Colombia) and Costa Rica comply with this requirement (Forman et al., 2014). 

Therefore, the present study aims to estimate the APC effects on the colorectal, colon and rectal cancer incidence in four Latin American areas covered by PBCR from 1983 to 2012 in Cali (Colombia); from 1983 to 2007 in Costa Rica; and from 1988 to 2012 in Goiânia (Brazil) and Quito (Ecuador).

## Materials and Methods

*Study design and population*

Ecological time series study with a population between 20 and 79 years old diagnosed with colorectal cancer reported in the PBCR from 1983 to 2012 in Cali, (Colombia, N= 5,538); from 1983 to 2007 in Costa Rica (N= 4,373); and from 1998 to 2012 in Goiânia (Brazil, N= 3852) and Quito (Ecuador, N= 2463).

*Data source*

Colorectal cancer data in each region, by year and age at diagnosis, and the population size were obtained from the series Cancer Incidence in Five Continents (CI5), volumes VI to XI, published by the International Agency for Research on Cancer (IARC)(Forman et al. 2014). However, we observed discrepancies of population size at risk from 2003 to 2007 in Goiânia, between the series CI5 and estimates from the “Instituto Brasileiro de Geografia e Estatística (IBGE)”. Thus, for this period, IBGE data were chosen.

CI5 data are obtained from high-quality cancer registries of a particular country or region(Parkin and Bray 2009). The four PBCRs were chosen by a rigorous editorial process which reached the highest level of quality and for being these registries in Latin America the ones with at least 20 years of uninterrupted time series.

All CI5-provided data are coded according to the International Classification of Diseases for Oncology, 3rd Edition (CID-O-3) and converted to the tenth edition (CID-10) with the following topographic codes: colorectal cancer (C18-21), colon cancer (C18) and rectal cancer (C19-21). This process ensures that the same validity checks are applied to all the data from different regions(Forman et al., 2014).

*Data Analysis*

For each region, crude and specific incidence rates by age group and sex were estimated for each 5-year period. Age-standardized incidence rates were calculated using the truncated method and world standard population (Segi et al., 1960; Doll et al., 1966).

APC models were estimated in order to discern the effects of age, period, and birth cohort on colorectal, colon and rectal cancer incidence rates. Age was grouped into 5-year intervals starting at 20-24 years until 75-79 years. The study periods were also grouped into 5-year intervals as follows: five periods for Goiânia, Quito (1988-1992 to 2008-2012) and Costa Rica (1983-1987 to 2003-2007) and six periods for Cali (1983-1987 to 2008-2012). Birth cohorts were estimated by subtracting the midpoint of the 5-year age group from the corresponding 5-year period.

APC effects with their respective rate ratios (RR) and 95% CI 95% confidence interval (95% CI) were calculated using the Poisson regression technique. The APC effects act multiplicatively on the rate, and its logarithm of the expected rate is a linear function of the effects of age, period, and cohort, given as (Doll et al., 1966; Holford, 1983; Ben-Shlomo and Kuh, 2002; Borges et al., 2018): 

$\ln\left(E\left[\mathrm{rji}\right]\right)=\ln\left(\frac{\mathrm{\theta ij}}{\mathrm{Nij}}\right)=\mu +\mathrm{\alpha i}+\mathrm{\beta i}+\mathrm{\gamma k}$

Where E[r*ij*] denotes the expected incidence in age group *i* and period *j*; ө*ij* the number of cases in age* i* and period *j*, and N*ij* the population at risk in age *i* and period *j*; µ is the average value of effects (intercept); α, is the effect of the age group *i*, β*j*,is the effect of time period *j* and *γ*k is the effect of cohort *k* (Holford 1983, 1991; Clayton and Schifflers, 1987).

The main problem to estimate the independent effects of age, period and cohort by APC analysis is the exact linear dependency among these factors and interferes on the estimation of the three effects using a full model, called nonidentifiability. In the current study, the parameterization method developed by Holford (1991) was chosen, since such method estimates APC effect parameters using deviations, curvature and drift as the estimable functions (Borges et al., 2018). This method was applied to allow us to interpret the period and cohort effects as a rate ratio (RR) relative to the reference cohort (1938, for Cali and Costa Rica and 1943, for Goiânia and Quito). The drift inclusion with cohort effect makes the age effect interpreted as the age-specifics rates in the reference cohort were adjusted by the period effect. The period effect function was set at zero average with zero slope, which is interpreted as the period related RR, after the adjustment to age and cohort.

Goodness of fit model was tested using the deviance, which was defined as two times the log-likelihood ratio of the estimated model compared with the full model. The contribution of the effects was tested by comparing the deviance of the specific effect model with the full model (APC). The findings were considered statistically significant at p<.05. APC analyses were performed using the statistical software R, version 3.5.1, Package Epi 2.0.

## Results

Between 1983-2012 in Cali, 5,528 patients with colorectal cancer were registered, of whom 44.3% were men and 3,080 55.7% were women. In Costa Rica, between 1983-2007, 8,595 patients were diagnosed with colorectal cancer, of whom 49.4% were men and 50.6%, women. From 1988 to 2012, Goiânia registered 3,856 cases of colorectal cancer, of whom 46.0% were men and 54.0%, women. In this same period, 2,463 patients with colorectal cancer were registered in Quito, of whom 44.2% were men and 55.8% were women.

*Colorectal cancer*

**Figure 1:**
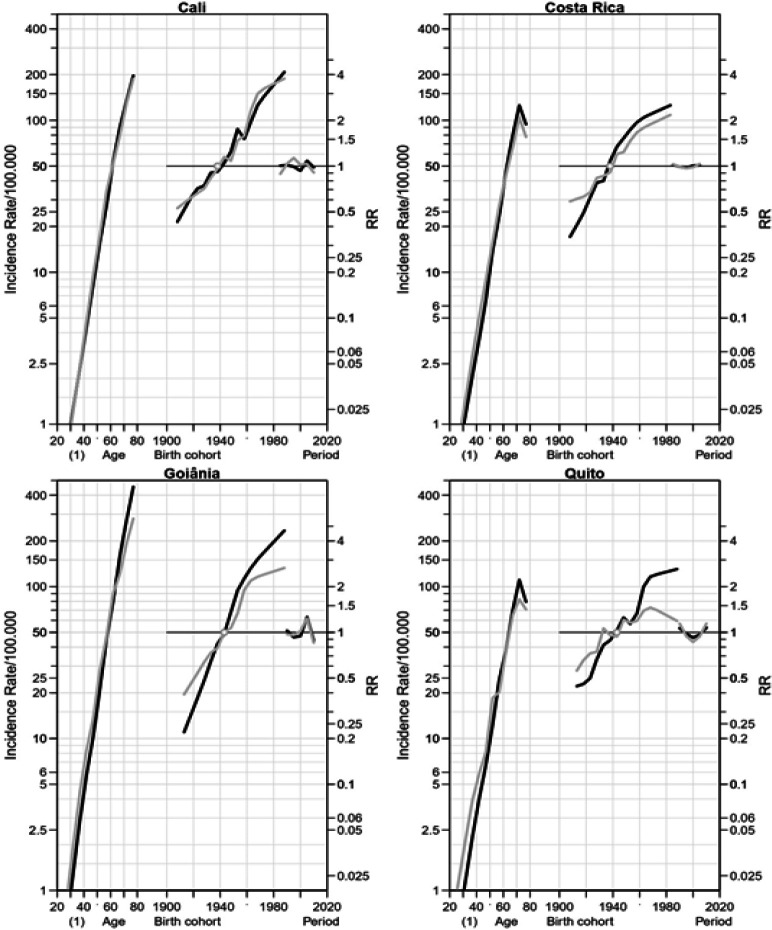
Age, Period and Cohort Effects on the Incidence of Colorectal Cancer among Women (Grey) and Men (Black) in Cali (Colombia) from 1983 to 2012, in Costa Rica from 1983 to 2007 and in Goiânia (Brazil) and Quito (Ecuador) from 1988 to 2012. RR Rate Ratio

Figure1 shows the contributions of age, period, and cohort to colorectal incidence rates. Age effect was statistically significant for both genders in all PBCR areas and the curves slope reached peaks in the older age groups. In Costa Rica and Quito, the peak was reached in the age group 70-74 years, while in Cali and Goiânia, the highest rates were observed in the age group 75-79 years. 

In all PBCR areas, the cohort effect was observed, except for women from Quito. An increased RR was observed in both gender from 1939 in Cali and Costa Rica and from 1943 in Goiânia. In Quito, a raised RR was found merely for men. Regarding the period effect, 2003-2007 presented an increased RR for both gender in Goiânia.

*Colon cancer*

**Figure 2:**
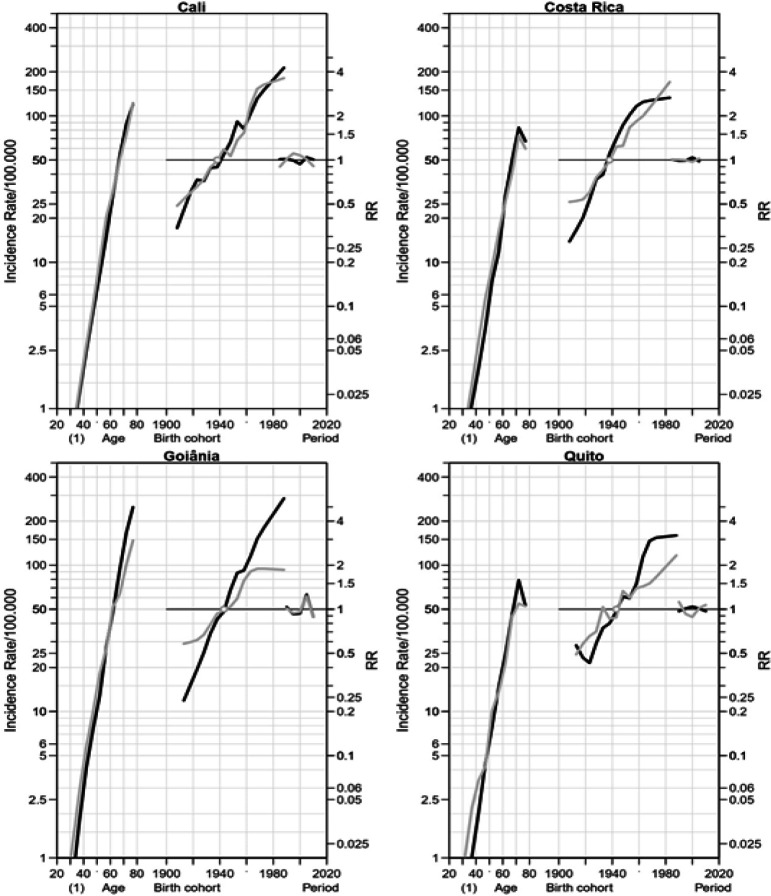
Age, Period and Cohort Effects on the Incidence of Colon Cancer among Women (Grey) and Men (Black) in Cali (Colombia) from 1983 to 2012, in Costa Rica from 1983 to 2007 and in Goiânia (Brazil) and Quito (Ecuador) from 1988 to 2012. RR Rate Ratio

Concerning to colon cancer, age effects were found for both genders in all PBCR areas (Figure 2). The cohort effect was identified for both gender born since 1939 in Cali and Costa Rica. In Goiânia, there was an increase in RR for men since 1943, while in women there was an increase in RR from 1943 to 1973, trending to steady afterwards. In Quito, for men the RR presented growth up to 1965 and after was stood stable. Among women, was identified an increased RR with a fluctuation at 1948-1953 period. Regarding to period effect, the same pattern was found as the colorectal cancer. 

*Rectum cancer*

**Figure 3:**
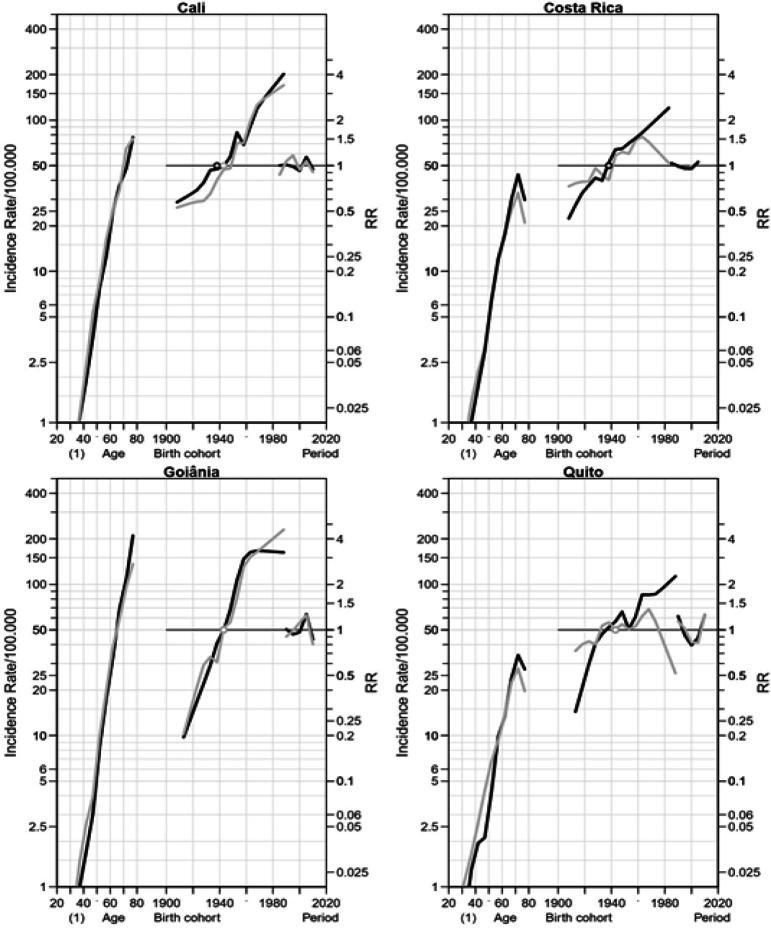
Age, Period and Cohort Effects on the Incidence of Rectum Cancer among Women (Grey) and Men (Black) in Cali (Colombia) from 1983 to 2012, in Costa Rica from 1983 to 2007 and in Goiânia (Brazil) and Quito (Ecuador) from 1988 to 2012. RR Rate Ratio

The APC effects for rectum cancer are displayed on Figure 3. Age effects were observed for rectal cancer in both genders of all PBCR areas. An increased cohort effect was observed for cohorts after 1953 of men and women in Cali, and after 1943 of men in Costa Rica, and after 1953 of women in Goiânia. In Goiânia men of cohort-birth from 1948 had a RR rise with stabilization after 1973. For men of Quito, an increased RR was observed for cohorts after 1963 with stabilization after 1973. An increased period effect was observed for men and women in Goiânia and Quito.

*Model Evaluation*

Table 1 illustrates the goodness of fit of APC models. For colorectal cancer the full model (APC) yielded a better fit for both gender in Goiânia, and for women in Cali and in Quito. However, the age-drift model showed a better fit for the other models. Regarding to colon cancer, the APC model showed a better fit for men in Goiânia and age-cohort model to men in Costa Rica whereas the age-drift model better fit all other PBCR areas. For rectum cancer in Goiânia and Quito, the APC model showed a better fit, while in Cali and Costa Rica the better fit was the age-drift model.

**Table 1:** Goodness of Fit of the Age-Period-Cohort Models for Colorectal Cancer, Colon Cancer and Rectum Cancer by Sex in Cali (Colombia) from 1983 to 2012, in Costa Rica from 1983 to 2007 and in Goiania (Brazil) and Quito (Ecuador) from 1988 to 2012

PCBR and Model	Colorectal cancer								Colon cancer								Rectum cancer							
	Men				Women				Men				Women				Men				Women			
	Resid df	Resid dev	Dev.	P*	Resid df	Resid dev	Dev.	P*	Resid df	Resid dev	Dev.	P*	Resid df	Resid dev	Dev.	P*	Resid df	Resid dev	Dev.	P*	Resid df	Resid dev	Dev.	P*
Cali																								
Age	63	179.099			63	196.062			64	140.709			64	127.012			63	111.674			63	120.381		
Age-drift	62	70.507	108.592	<0.0000	62	98.922	97.14	<0.0000	63	65.209	75.5	<0.0000	63	69.04	57.972	0	62	77.026	34.648	0	62	81.255	39.126	0
Age-Cohort	53	57.165	13.342	0.1477	53	81.044	17.878	0.0366	54	56.315	8.894	0.4471	54	54.402	14.638	0.1014	53	69.255	7.771	0.5574	53	71.652	9.603	0.3836
Age-period-cohort	50	51.289	5.876	0.1178	50	66.894	14.15	0.0027	51	54.849	1.466	0.6901	51	46.86	7.542	0.0565	50	62.966	6.29	0.0983	50	61.453	10.199	0.0169
Age-period	59	65.164	-13.874	0.1269	59	87.612	-20.718	0.1396	60	64.048	-9.199	0.4191	60	61.677	-14.817	0.0961	59	70.687	-7.721	0.5624	59	73.495	-12.042	0.2109
Age-drift	62	70.507	-5.343	0.1483	62	98.922	-11.31	0.0101	63	65.209	-1.161	0.7624	63	69.04	-7.363	0.0612	62	77.026	-6.339	0.0962	62	81.255	-7.76	0.0512
Costa Rica																								
Age	51	212.087			51	141.946			51	200.099			51	132.942			51	74.024			51	73.981		
Age-drift	50	45.969	166.118	<0.0000	50	64.375	77.571	<0.0000	50	60.597	139.502	0	50	57.157	75.785	<0.0000	50	38.868	35.156	0	50	64.088	9.892	0.0017
Age-Cohort	42	30.889	15.08	0.0576	41	53.228	11.147	0.2658	42	44.802	15.795	0.0454	41	50.823	6.333	0.7062	41	34.248	4.62	0.8661	41	51.707	12.381	0.1927
Age-period-cohort	40	30.221	0.667	0.7162	39	51.244	1.984	0.3708	40	43.698	1.104	0.5757	39	50.066	0.757	0.6848	39	32.117	2.131	0.3445	39	50.319	1.389	0.4994
Age-period	48	45.547	-15.325	0.0531	48	60.575	-9.331	0.4073	48	59.011	-15.313	0.0533	48	55.464	-5.398	0.7983	48	37.154	-5.037	0.8311	48	62.116	-11.797	0.225
Age-drift	50	45.969	0.422	0.8097	50	64.375	-3.8	0.1496	50	60.597	-1.586	0.4525	50	57.157	-1.692	0.429	50	38.868	-1.714	0.4245	50	64.088	-1.973	0.3729
Goiania																								
Age	51	245.744			51	166.188			51	154.302			51	91.41			51	125.515			51	138.893		
Age-drift	50	96.402	149.342	<0.0000	50	100.812	65.376	0	50	72.341	81.961	0	50	68.401	23.009	0	50	57.709	67.806	0	50	92.088	46.806	0
Age-Cohort	42	86.87	9.531	0.2995	42	88.702	12.11	0.1464	42	67.707	4.634	0.7959	42	63.989	4.412	0.8181	42	47.394	10.314	0.2436	42	73.583	18.504	0.0177
Age-period-cohort	40	47.024	39.847	<0.0000	40	46.094	42.608	0	40	44.625	23.082	0	40	43.454	20.534	0	40	29.822	17.572	0.0001	40	47.369	26.214	0
Age-period	48	54.675	-7.651	0.4683	48	56.634	-10.54	0.2291	48	48.249	-3.624	0.8894	48	48.153	-4.699	0.7892	48	39.949	-10.126	0.2563	48	62.986	-15.617	0.0482
Age-drift	50	96.402	-41.727	<0.0000	50	100.812	-44.178	0	50	72.341	-24.092	0	50	68.401	-20.248	0	50	57.709	-17.76	0.0001	50	92.088	-20.101	0
Quito																								
Age	51	121.667			51	114.821			51	94.645			51	117.151			51	94.175			52	79.734		
Age-drift	50	72.429	49.238	0	50	92.505	22.315	0	50	66.652	27.992	0	50	96.846	20.306	0	50	72.805	21.37	0	51	76.108	3.627	0.0568
Age-Cohort	40	64.499	7.93	0.6357	40	81.613	10.892	0.2831	40	59.577	7.075	0.7183	41	86.331	10.514	0.3105	42	68.936	3.869	0.8687	41	68.726	7.382	0.6889
Age-period- cohort	38	61.387	3.112	0.211	39	66.979	14.634	0.0006	38	59.2	0.377	0.8383	39	81.769	5.563	0.1021	40	56.722	12.214	0.0022	39	53.188	15.537	0.0004
Age-period	48	69.549	-8.161	0.6131	48	79.342	-12.363	0.1936	48	66.553	-7.352	0.6918	48	92.852	-11.083	0.27	48	64.048	-7.326	0.5018	49	60.387	-7.198	0.7066
Age-drift	50	72.429	-2.881	0.2369	50	92.505	-13.163	0.0013	50	66.652	-99	0.9515	50	96.846	-3.994	0.1357	50	72.805	-8.757	0.0125	51	76.108	-15.721	0.0004

Abbreviations: PBCR, population-based cancer registry; Resid Dev, residual deviance for the Poisson model; Resid df, degrees of freedom from deviance residue; *P value based on the x^2^ test comparing the two-factor model (age-period or age-cohort) and the complete model (age-period-cohort)

**Table 2:** Crude Incidence Rates by Age Group in Goiania (Brazil) from 1988 to 2012

Subsite	Age group	Crude rate (Men)					Crude rate (Women)				
		1988-1992	1993-1997	1998-2002	2003-2007	2008-2012	1988-1992	1993-1997	1998-2002	2003-2007	2008-2012
Colorectal cancer	20-24	0.9	0.0	1.1	1.9	0.6	1.1	0.4	0.6	0.9	0.0
	25-29	1.4	1.8	1.2	2.9	1.6	3.4	0.4	1.8	4.3	2.0
	30-34	3.4	2.0	4.6	7.0	4.8	5.5	3.5	4.4	7.6	4.7
	35-39	4.1	6.0	7.7	1.1	6.2	3.6	8.3	9.7	9.2	8.5
	40-44	10.2	10.9	9.6	14.3	16.3	6.9	11.0	16.5	24.1	18.4
	45-49	19.8	12.3	16.7	31.5	21.5	10.1	17.0	19.5	30.3	25.0
	50-54	15.4	15.8	27.2	47.8	47.3	27.6	24.7	32.6	46.6	44.4
	55-59	18.7	30.9	47.3	79.0	80.2	35.8	40.0	46.0	57.8	55.0
	60-64	46.9	45.0	79.0	88.4	95.4	52.1	50.0	63.4	129.1	84.4
	65-69	47.2	71.8	102.9	177.7	135.3	51.5	83.0	113.8	113.7	95.9
	70-74	113.6	95.4	142.3	212	189.7	98.2	117.2	119.1	161.8	135.7
	75-79	80.0	146.6	109.1	271.9	287.3	121.9	79.9	166.7	221.4	181.1
Colon cancer	20-24	0.4	0.0	0.4	0.9	0.3	0.8	0.4	0.3	0.6	0.0
	25-29	0.5	1.3	0.8	1.6	0.9	2.5	0.4	0.4	1.8	0.9
	30-34	0.6	1.0	1.8	4.7	3.1	2.5	0.9	1.6	5.5	1.9
	35-39	2.1	3.6	4.1	9.8	4.1	1.8	5.7	3.5	5.6	4.1
	40-44	7.6	7.3	6.6	8.9	11.2	6.1	4.5	11.3	12.5	12.0
	45-49	13.2	9.5	9.1	20.4	13.1	5.0	11.9	13	17.4	15.4
	50-54	11.2	4.9	15.6	29.4	23.3	22.3	18	19.7	24.8	23.2
	55-59	13.1	19.5	29.7	48.4	44.6	24.4	21.4	21.2	33.0	31.0
	60-64	37.1	23.6	49.2	49.9	55.9	33.3	33.9	44.7	71.0	45.6
	65-69	21.8	40.6	60.3	103.5	82.8	28.6	43.9	59.8	67.3	55.3
	70-74	73.8	76.3	91.2	132.8	112.5	80.3	55.4	65	84.9	87.8
	75-79	35.6	92.6	66.7	131.4	186.7	94.8	51.3	98.3	129.2	101.2
Rectum cancer	20-24	0.4	0.0	0.7	0.9	0.3	0.4	0.0	0.3	0.3	0.0
	25-29	1.0	0.4	0.4	1.3	0.6	0.8	0.0	1.5	2.5	1.2
	30-34	2.8	1.0	2.7	2.3	1.7	3.0	2.6	2.8	2.2	2.8
	35-39	2.1	2.4	3.6	6.3	2.1	1.8	2.6	6.2	3.6	4.5
	40-44	2.6	3.7	3.0	5.4	5.1	0.8	6.5	5.2	11.6	6.4
	45-49	6.6	2.8	7.6	11.1	8.4	5.0	5.1	6.5	12.9	9.7
	50-54	4.2	10.9	11.7	18.4	24.0	5.3	6.7	12.9	21.7	21.2
	55-59	5.6	11.4	17.6	30.6	35.6	11.4	18.6	24.8	24.8	23.9
	60-64	9.9	21.5	29.9	38.5	39.5	18.7	16.1	18.7	58.2	38.8
	65-69	25.4	31.2	42.7	74.2	52.5	22.9	39	54	46.4	40.6
	70-74	39.8	19.1	51.1	79.1	77.2	17.9	62.8	54.1	76.8	47.9
	75-79	44.4	54.0	42.4	140.5	100.6	27.1	28.5	68.4	92.3	80

## Discussion

The APC model to colorectal, colon and rectum cancer incidence showed an age effect on the increase incidence rates for both gender in all PBCR areas and the curves slope reached peaks in the older age groups. Lifetime accumulated exposures may be a hypothesis for this scenario due to the amount as well as duration of exposures that is accumulating gradually over life, damaging biological systems (Ben-Shlomo and Kuh, 2002).

Although incidence rates were influenced by age effect, some differences were observed among PBCR areas. Whereas Cali and Costa Rica reached the peak of the incidence rate at 70-74 years age group, Goiânia and Quito presented the highest rates at 75-79 years age group. These differences could reflect later diagnosis as a result of difficulties in health care services access besides the lack of screening programs (Oliveira et al., 2018). A Brazilian study (Souza et al., 2016) aiming to analyze associated factors relating to colorectal cancer late diagnosis in Public Health System (SUS) users, found 52.5% of late diagnosis of which 83.2% reported difficulties in health care access. 

The cohort effects in our study suggest that economic market opening that occurred in Latin America countries at the late 80’s to beginnings 90’s (Béjar et al., 2011) as the main fact that partly explained the changes in risk factors exposures, especially those related to dietary and lifestyle in the younger cohorts (Popkin, 2004; Eaglehouse et al., 2017). There has been a gradually replacement in standard consumption, reducing unprocessed or minimally processed foods intake and raising of ready-to-eat or ready-to-heat ultra-processed food products (Monteiro et al., 2011). These hypotheses are supported by evidences suggesting positive association between colorectal cancer with red and processed meat intake, obesity, alcoholic beverages and smoking (Johnson et al., 2013; Bouvard et al., 2015). Conversely, physical activity and vegetables and fruits consumption were considered as protector factors. Similar finding were reported by Kok et al., (2008) in the colorectal cancer incidence of Singapore.

In addition to diet changes, economic prosperity and technology advances have also led to increased sedentary behavior (Eaglehouse et al., 2017). Hallal et al., (2014) performed sectional studies in 2002, 2007 and 2012 to estimate the prevalence of physical activity in Pelotas, Brazil. The short version of International Physical Activity Questionnaire was used to assess the prevalence of physical inactivity defined as less than 150 min/week. The prevalence of physical inactivity was found at 41.1% (95% CI: 37.4–44.9) in 2002, at 52.0% (95% CI: 49.1–53.8) in 2007, and at 54.4% (95% CI:51.8–56.9) in 2012 (p<0.001). Thus, the physical inactivity pattern might have influenced the cohort effect observed in the studied regions.

Although cohort effects were observed, differences were found among anatomic sites probably due to distinct etiological factors (Eaglehouse et al. 2017). Wei et al., (2004) used data from two prospective cohorts studies to evaluate the association of risk factors and development of colon and rectum cancer. Authors observed that age, sex, family history, height, BMI, physical activity, alcohol, red and processed meat being related to colon cancer, whereas age and gender showed association with rectal cancer (Wei et al., 2004). These findings suggest that risk factors seems not to contribute equally for all anatomic sites (Eaglehouse et al., 2017).

Period effect was observed in our study for both genders and colon and rectum sites in Goiânia, with increased rate ratios between 2003-2007, decreasing afterwards. In 2002, Norms and Guidelines for colorectal cancer prevention were published by Instituto Nacional de Cancer José Alencar Gomes da Silva (INCA) (INCA 2003). This improvement on medical surveillance may have increased prevalent cases detection. Giorgi Rossi et al., (2015) reported the impact of Italian colorectal cancer screening program. Authors suggested that prevalent lesions detection led to raise of colorectal cancer rates in target population, 50-69 age groups.

In the present study, an increasing incidence of colorectal, colon and rectal cancer was observed in Goiânia, being remarkable in the period 2003-2007 in ≥50 age groups (Table 2). The Norms and Guidelines published in Brazil also included other cancers (INCA 2002, 2016) which raised medical surveillance for malignant tumors. This opportunistic screening also seems to have contributed to the growth of colorectal cancer detection reflected by the noticed period effect.

Limitations of the present study include the use of secondary data in the analysis. However, the selected PBCR provided high quality standardized information. Besides that, our results are consistent to other population-based study’s findings. Another limitation would be the fact that this study is not representative of all Latin America countries, because data were analyzed from of cities’ PBCR including Goiânia, Cali, Quito. Only the PBCR of Costa Rica covers the whole country. On the other hand, one of the strengths of this study is that to our knowledge, the current study is the first to use APC modeling to examine trends in colorectal, colon, and rectum cancer incidence using data until 2012 from different regions of Latin America. Furthermore, APC modeling represents a comprehensive examination of the age, period and birth cohort effects, pointing out which effects had influenced the colorectal, colon, and rectal incidence trends.

In conclusion, the present study showed an increasing trend in colorectal cancer for both genders over the past 30 years in Cali, and in the past 25 years in Costa Rica, Goiânia, and Quito. This increase seems to be mainly a result of cohort effects, suggesting that the opening of economic market process led to changes in the risk factors exposures in those countries, such as those changing in diet (increasing access of processed and ultra-processed food) and lifestyle (sedentary lifestyle), might be some possible explanations. Furthermore, the period effect observed in Goiânia appears to reflect the effect of the implementation of norms and guidelines for colorectal cancer prevention and of screening programs for breast, cervical, and prostate cancer that could in part explain the rise of the colorectal cancer incidence. Thus, such knowledge provided a better comprehension of specific factors affecting the colon and rectum cancer trends in those countries, shedding light on the strategies necessary for prevention, early detection and control of those cancers in each studied countries.
